# Abnormal Placenta

**Published:** 1856-05

**Authors:** J. R. Johnson

**Affiliations:** Lebanon, Ala.


### ARTICLE III.

*Abnormal Placenta.* By Dr. J. R. Johnson, Lebanon, Ala.

The following case may not be without some interest, and is therefore respectfully submitted for publication, with the hope that it may elicit some explanation from the profession, and add to the meagre stock of facts in this particular department of science.

I have been unable to find even in Churchill—who is remarkable for embracing a profusion of interesting obstetrical facts—any account of a similar deviation, and I therefore present it, in connection with a brief history of the case in which it was observed.

On the 7th of January, I was summoned to see Mrs. B——, aged 42 years, the mother of eight or nine children, rather corpulent, and enjoying general good health; found her in the eighth month of utero-gestation, with labor pains and uterine hemorrhage; upon examination, found the os tincæ dilated to about the size of a twenty-five cent coin; presentation natural; parts relaxed, and an apparently favorable condition, with the exception of hemorrhage. I remained during the night, and on the morning of the 8th left her; the labor pains and hemorrhage having both subsided under treatment directed to the latter. I found it necessary to visit her again on the 9th, at 8'clock, P. M., when I found the labor in the second stage, and in about fifteen minutes, she was delivered of a living child, which was followed by considerable hemorrhage; this continuing, and feeling some apprehension for the life of my patient, after manipulation, friction, and the application of cold cloths over the uterine region, without the effect of dislodging the placenta, or arresting the hemorrhage, I introduced my hand, and found the placenta only partially detached; more powerful contractions, however, being induced by the presence of the hand, I succeeded in separating the adherent portion, and with it, or near by, a *tumor* or *second* placenta, apparently attached to the uterine walls, and differing but little in its appearance from the placenta proper, except in its size, and the want of a well defined funis, being about one third the size of the *true placenta*, and connected with the decidious membrane, some six inches from it. Hemorrhage ceased, and no further difficulty occurred.

